# β_2_-Adrenergic Receptor Agonists in Diabetic Kidney Disease: Exploring a New Frontier

**DOI:** 10.1155/bri/5428052

**Published:** 2025-06-19

**Authors:** Shreya Hegde, Bharti Chogtu, Rahul Magazine, Ravindra Prabhu

**Affiliations:** ^1^Department of Pharmacology, Kasturba Medical College, Manipal Academy of Higher Education, Manipal, Karnataka 576104, India; ^2^Department of Respiratory Medicine, Kasturba Medical College, Manipal Academy of Higher Education, Manipal, Karnataka 576104, India; ^3^Department of Nephrology, Kasturba Medical College, Manipal Academy of Higher Education, Manipal, Karnataka 576104, India

**Keywords:** β_2_-adrenergic agonists, diabetic kidney disease, fibrosis, mitochondrial biogenesis, renal inflammation

## Abstract

Diabetic kidney disease is a major cause of end-stage kidney disease. Various metabolic, hemodynamic, inflammatory, and profibrotic factors secondary to diabetes mellitus result in complex intracellular signaling, which in turn is responsible for the functional and structural changes associated with diabetic kidney disease. The beneficial effects of β_2_-adrenergic agonists on renal cells bearing β_2_-adrenergic receptors in diabetic kidney disease models have been reported. This narrative review explains the various mechanisms by which β_2_-adrenergic agonists can have potential beneficial effects on diabetic kidney disease and highlights various in vitro, animal and human studies which lend credence to this hypothesis. It also touches upon the challenges and future concerns regarding their use in patients with this condition.

## 1. Introduction

Adrenergic receptors (ARs), which serve as binding sites for catecholamines, are widely distributed in the body and play important roles in diverse physiological processes. These receptors are broadly classified into alpha (α) and beta (β) receptors on the basis of their physiological specificities and pharmacological effects [1]. Within this group, α-ARs are further classified as α1 and α2, whereas β-ARs are divided into β1, β2, and β3 [2]. Similar to all G-protein-coupled receptors, the β2 receptor consists of 7 transmembrane α-helices. It features three extracellular loops, including the amino terminus, and three intracellular loops, ending with a carboxy terminus [3]. Although β_2_ receptors are predominantly located in airway smooth muscles, they are also found in a variety of other tissues, including cardiac muscles, uterine muscles, mast cells, mucous glands, epithelial cells, vascular endothelium, eosinophils, and skeletal muscles [4]. The presence of β_2_ receptors on different types of renal cells has also been confirmed via in vitro autoradiography. Whereas β_1_ receptors are predominantly found in the glomeruli and juxtaglomerular cells of the cortex, β_2_ receptors are localized in the medullary tubules [5]. Through immunohistochemistry using rabbit antibodies in the rat kidney, β_2_ receptors were found predominantly in the apical membrane of proximal convoluted tubules [6]. Given the presence of β_2_ receptors in renal tissues, few in vitro and animal studies have investigated the role of β_2_ receptors in preventing the development and progression of chronic kidney disease (CKD). Diabetic kidney disease (DKD) is the most common cause of end-stage renal disease [7], and this review explores the beneficial role of β_2_-AR agonists in DKD and summarizes the different mechanisms by which these drugs act, as postulated by various studies.

### 1.1. Diabetic Kidney Disease and Its Pathophysiology

The glomerulus, the filtering unit of the kidney, consists of capillaries situated between resistance vessels. These specialized capillaries are formed by the first layer of fenestrated endothelium on the luminal side, the second layer of the glomerular basement membrane, and the distal layer of visceral epithelial cells or podocytes. Podocytes, in addition to providing supporting structures with mesangial cells, form a filtration barrier along with endothelial cells and the basement membrane [8].

Podocytes do not have regenerative capacity, and dysregulation of their growth and differentiation is the characteristic of DKD. Glomerular hyperfiltration, glomerulomegaly, podocyte foot process effacement, podocyte hypertrophy, podocyte loss in the late stage, glomerular scarring, proteinuria, and increased expression of the vascular endothelial growth factor (VEGF) are characteristic features of DKD [9].

The prevalence of CKD among individuals with type 2 diabetics ranges from 27% to 29% [10, 11]. DKD refers to “the clinical diagnosis of kidney disease attributed to diabetes based on the presence of albuminuria (> 300 mg/d) and/or the low estimated glomerular filtration rate (eGFR) (< 60 mL/min/1.73 m^2^) in patients with type 2 diabetes for more than 3 months, but an even lower threshold of albuminuria can signify DKD in patients with type 1 diabetes.” [9] The latest Kidney Disease: Improving Global Outcomes (KDIGO) guidelines use the term ‘diabetes and kidney disease' instead of ‘diabetic kidney disease' to avoid suggesting that CKD always stems from typical diabetes-related causes in all cases. The term diabetic kidney disease can still be used, as long as this distinction is clear (KDIGO Diabetes Work Group [12]).

The pathogenesis of DKD can be attributed to four classes of causal factors: metabolic, hemodynamic, growth, and proinflammatory factors [13]. In diabetes mellitus, hyperglycemia causes kidney damage by inducing glomerular hyperfiltration and hypertension [14]. Various vascular and tubular factors in diabetes mellitus cause a net increase in efferent arteriolar resistance and a net decrease in afferent arteriolar resistance, leading to an increase in the glomerular filtration rate [15]. Hyperglycemia triggers the formation of advanced glycation end products (AGEs), reactive oxygen species (ROS), and the activation of the protein kinase C (PKC) and JAK-STAT pathways [16]. The JAK-STAT pathway plays an important role in the development of DKD, and in particular, the JAK2/STAT 3 pathway is upregulated in both animal models and DKD patients. The renin angiotensin system, fibrosis, the immune response, the inflammatory response, senescence, injury, and autophagy upregulate various cytokines and growth factors by activating the JAK-STAT pathway in DKD [17]. PKC is a family of serine/threonine kinases that can bind to diacylglycerol (DAG) or Ca^++^ and positively regulate kinase activity. Hyperglycemia activates PKC and increases connective tissue growth and transforming growth factor (TGF)-β, which leads to mesangium expansion and nephromegaly, and this in turn results in glomerulosclerosis and diabetic nephropathy [18]. Additionally, increased production of DAG activates PKC, and AGEs and ROS also activate PKC. AGEs increase the expression of serum amyloid A, which perpetuates inflammatory gene expression, leading to fibrosis. Thus, hyperglycemia is responsible for promoting inflammation and fibrosis [19]. The VEGF regulates vascular permeability and angiogenesis and plays an important role in diabetic nephropathy. The VEGF is activated early in the kidneys, leading to vascular expansion, which in turn causes hyaline arteriosclerosis and hypertensive changes [20]. Hyperglycemia also alters podocyte metabolism by inducing dynamin-related protein 1 (Drp1)-mediated mitochondrial fission triggered by ROCK-1 [21].

In view of the abovementioned changes produced by increased blood glucose levels, preventive strategies for DKD are necessary. Prevention of DKD involves multiple strategies, and prescribing appropriate medication at an early stage is one of them [10]. Angiotensin-converting enzyme inhibitors, angiotensin receptor blockers, and sodium glucose transport protein 2 (SGLT-2) inhibitors are currently approved drugs for treating DKD. However, these drugs do not reverse the disease process. They are involved in slowing the progression of the pathology. Other drugs actively studied for DKD treatment include mineralocorticoid receptor antagonists; nuclear factor erythroid 2-related factor 2 activators, such as bardoxolone methyl; hypoxia-inducible factor prolyl hydroxylase inhibitors; glucagon-like peptide 1 receptor agonists; dipeptidyl peptidase-4 inhibitors; AGE inhibitors; and epigenetic regulators. It has been suggested that evaluating drugs that target podocytes, immune cells, and fibroblasts is desirable [9]. Among this myriad of possible targets, β2 agonism is emerging as one of the potential mechanisms for addressing the pathophysiology of DKD [22]. A recent study in β_2_-AR knockout mice revealed that β_2_ receptors are critical for the recovery mechanism and that drugs targeting these receptors may be useful in treating podocytopathies [23].

### 1.2. Role of β_2_ Receptors in Renal Physiology

Beta receptors are distributed across various tissues in the body.  Table 1 highlights their localization and function of these receptors in the kidneys.

β_2_ receptors modulate renal hemodynamics, inflammation, TNF-α production, and cell death [27]. In proximal tubules, β_2_-ARs mediate increases in Na-K-ATPase activity and sodium flux via transducer phosphokinase C (PKC) and thereby increase sodium entry into the apical membrane [28]. Prejunctional β-ARs in the kidney, which are present on sympathetic terminals, are of the β_2_ subtype and are associated with neurotransmission in the vascular bed [29].

### 1.3. Effects of β_2_-AR Agonists on Renal Inflammation and Fibrosis

Inflammation and fibrosis play significant roles in the pathogenesis of DKD. The anti-inflammatory effects of β_2_-agonists are mediated by the second messenger cAMP through protein kinase A (PKA) signaling. These target processes include restoring cytosolic calcium in neutrophils, inhibiting NF-kappa B, increasing IL-10, inhibiting lipopolysaccharides, and attenuating cytokine release by macrophages [30]. β_2_-Agonist activity is also mediated through Epac, which is a target of cAMP (second messenger) [31]. Overall, coupling of β_2_-AR with the stimulatory pathway Gs activates PKA and Epac and is responsible for the protective effects. On the other hand, the coupling of β_2_-AR with the inhibitory Gi or β arrestin pathway activates MAP kinases and is responsible for various deleterious effects [30].

A strong correlation between the extent of macrophage infiltration and the later development of tubular interstitial fibrosis, as well as the progression of DKD, has been reported [32, 33]. The degree of monocyte/macrophage accumulation is correlated with the severity of kidney damage, and β_2-_AR agonists have been shown to reduce monocyte activation [34].

The proinflammatory mediator TNF-α contributes to renal injury in diabetes. β_2_-AR agonists inhibit phorbol myristate acetate (PMA)-induced TNF-α in rat bone marrow macrophages. The β_2_-AR agonists metaproterenol and terbutaline prevent lipopolysaccharide-induced TNF-α production [34].

β_2_-AR inhibits profibrotic processes such as fibroblast proliferation and differentiation by increasing cAMP levels [35]. The antifibrotic activity of the long-acting β_2_-AR agonist olodaterol has been shown to be mediated by attenuation of proliferation, transdifferentiation, migration, contraction, extracellular matrix production, and profibrotic mediator release in human lung fibroblasts [36].

Sirtuin-1 (SIRT-1) is a nicotinamide adenine dinucleotide (NAD)-dependent histone deacetylase that plays an important role in protecting cells from ROS. Interventions to increase the activity of SIRTs can promote efficient cellular function. β_2_-AR agonists, by activating the cAMP/PKA pathway, also increase SIRT-1 activity [37].

### 1.4. Effect of β_2_-AR Agonists on Mitochondrial Biogenesis (MB)

**Figure 1:**
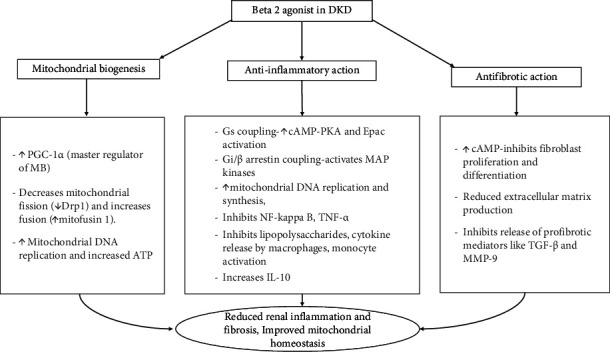
The role of β_2_-AR agonists in DKD.

Kidneys, which are highly metabolic, depend on mitochondrial oxygen consumption for the energy demands of tubular reabsorption [38]. Mitochondrial dysfunction in kidneys is a significant event in the initial stages of diabetes [39]. In addition to their anti-inflammatory effects, β_2_-AR agonists activate MB, which maintains high-energy demands and metabolic homeostasis following injury. This process increases the mitochondrial copy number and ATP output and can occur under basal conditions [40]. Peroxisome proliferator-activated receptor gamma coactivator 1 alpha (PGC1α) is a transcriptional coactivator that regulates MB by controlling mitochondrial energy metabolism [41]. PGC-1α is regulated by nuclear receptors of the PPAR family, and clinical trials have shown that agonists of PPARα and γ induce antialbuminuric effects [42]. PGC-1α is downregulated in proximal tubules in animal models of diabetes and thus leads to disease progression. MB preserves homeostasis under physiological and pathological conditions in mitochondria and plays a critical role in repair and recovery after kidney injury [43]. In view of these findings, MB has been identified as a potential therapeutic target that can ameliorate the mitochondrial dysfunction observed in various systemic disorders [44]. Researchers have demonstrated that β_2_-AR agonists such as formoterol improve mitochondrial dynamics and energetics in DKD [45]. High glucose levels increase the level of the mitochondrial fission protein Drp1 and decrease the level of the mitochondrial fusion protein mitofusin (Mfn1), resulting in an imbalance of proteins related to mitochondrial dynamics in renal proximal tubular cells and causing the progression of kidney disease [45, 46]. By acting on β_2_-ARs, formoterol promotes MB via various pathways to decrease mitochondrial fission and increase fusion, thereby maintaining mitochondrial homeostasis in renal proximal tubular cells [47]. To summarize, the role of β_2_-AR agonists in DKD can be categorized as an effect on MB, anti-inflammatory, and antifibrotic action (Figure 1).

## 2. Studies Suggesting the Role of β_2_-AR Agonists in Chronic Kidney Disease

### 2.1. In Vitro and Animal Studies

In vitro and animal studies have shown that the injury caused to kidney glomeruli and podocytes by diabetic nephropathy can be reversed by treatment with a β_2_-AR agonist, specifically formoterol. Additionally, studies have confirmed that formoterol is an inducer of MB and prevents mitochondrial dysfunction. The beneficial mechanisms of β_2_-AR agonists postulated in various studies are presented in Tables 2 and 3.

### 2.2. Human Studies

The evidence for the favorable effect of β_2_-AR agonists in DKD in humans has been documented in observational studies. To the best of our knowledge, no randomized controlled trials have evaluated the role of β_2_-AR agonists in DKD.

A study to evaluate the role of β_2_-AR agonists in preventing micro- and macrovascular complications in diabetic patients was conducted in Korea. In 2004, newly diagnosed diabetic patients were divided into two groups on the basis of β_2_-AR agonist intake and were observed until 2015. Vascular complications decreased with increasing duration of intake of β_2_-AR agonists in this study. Analysis of individual complications revealed a decrease in the hazard ratio as the duration of intake of β_2_-AR agonists increased. The protective effects of β_2_-AR agonists are most powerful against renal complications. The authors concluded that prospective controlled studies are warranted to confirm these results [52].

Another retrospective study of 24,133 veterans with CKD stage 4 revealed a significant decrease (25.6%) in progression to end-stage renal disease in chronic obstructive pulmonary disease (COPD) patients on β_2_-AR agonists compared with those without COPD or with COPD on drugs other than β_2_-AR agonists [51].

### 2.3. Role of β Blockers in DKD

Studies have shown opposing roles of β blockers in DKD. β_2_-AR agonists have shown protective effects in animal models and human studies. The nonselective β-blockers such as propranolol reduce renal perfusion by lowering cardiac output and renal perfusion pressure. This leads to stimulation of α1-mediated vasoconstriction and blocking β_2_-mediated vasodilation. On the other hand, cardioselective and vasodilatory beta blockers slow deterioration of renal function by preventing decrease in GFR. Carvedilol by its antioxidant action also decreases albuminuria [53]. A number of single-nucleotide polymorphisms (SNPs) of the *β AR subtypes* have been reported in cardiovascular diseases (CVDs) [54]. The Arg16Gly polymorphism in the β_2_-AR gene has been linked to variations in renal function, specifically eGFR. Individuals with the Gly16Gly variation had lower eGFR implicating impaired renal function [55]. The paradoxical responses by cardioselective and vasodilatory can be attributed to polymorphism of β_2_-ARs.

### 2.4. Challenges in Long-Term Systemic Therapy With β_2_-AR Agonists

The safety and effectiveness of β_2_-AR agonists in humans are well established in bronchial asthma and COPD. A study assessing the role of physical activity in CKD patients has shown that 28% of patients had COPD as a comorbidity [56]. β_2_-AR agonists can also be used as a bronchodilator in such patients. However, there are specific issues associated with β_2_-AR agonist therapy. During the initial few weeks of treatment, tachycardia can occur. Tremors, encountered at higher doses of formoterol, are also among the early symptoms reported, but tolerance to this effect eventually develops. In long-term therapy, tolerance also leads to adverse metabolic effects, such as increased plasma glucose due to increased glycogenolysis. Although negligible, this effect may be of concern when used in diabetic patients [57, 58]. An association between the use of β_2_-AR agonists and cardiac events such as arrhythmias, cardiac failure, and ischemia has been reported in some studies [59, 60]. These effects are more related to the older generation of β-agonists, such as fenoterol or isoproterenol [60]. The long-term systemic administration of formoterol is associated with cardiovascular effects such as cardiomyocyte hypertrophy, interstitial fibrosis, and diastolic dysfunction [60–62]. Formoterol has shown good cardiovascular safety in several studies, and in most of these studies, it was administered through the inhalational route [63–65].

### 2.5. Route of β_2_-AR Agonist Delivery in Kidney Disease

The side effects of β_2_-AR agonists prompted the search for an effective means to deliver the drug, mainly to the kidney, with minimal effects on other organ systems. Mesoscale nanoparticles (400–500 nM in diameter), which selectively localize themselves in the proximal tubules of the kidney, are being tried for drug delivery in renal diseases [66]. Formoterol encapsulated in poly (ethylene glycol) methyl ether-block poly(lactide-co-glycolide) nanoparticles induced MB in proximal tubules in kidneys. The formoterol-containing nanoparticles provided renal protection against ischemia/reperfusion injury in mice at a reduced dose and circumvented adverse cardiac effects [67, 68].

### 2.6. Role of β2-AR Agonist in Relation to CKD Staging

A poor agreement between chronic histological kidney damage and CKD staging has been observed. About 30%–40% CKD stage 3 patients had mild or no lesions on histological evaluation; however, 7%–10% of cases with CKD stage 1 had moderate or even severe histological lesions [69]. β_2_-AR agonist by their anti-inflammatory, antifibrotic, and modulating MB has shown a promising role in DKD. But to determine the CKD stage where it will be most beneficial cannot be clearly established as there is a poor agreement between histological changes and CKD staging.

## 3. Conclusion

β_2_-AR agonists seem to have potential benefits in DKD, as shown in in vitro, animal and human studies. Multiple mechanisms at different levels have also been postulated for these beneficial effects. However, clinical trials are warranted to confirm the status of β_2_-AR agonists in the prevention and treatment of DKD.

## Figures and Tables

**Table 1:** Localization and physiological functions of beta receptor subtypes in the kidney [24–26].

Type of beta receptors	Localization in the kidney	Physiological function
β_1_	Smooth muscles of juxtaglomerular cells	Renin release and subsequent activation of the renin angiotensin aldosterone system. Ultimately important role in renal sodium handling and BP regulation.
	Proximal and distal tubular segments and acid-secreting type A intercalated cells of the cortical and medullary collecting ducts.	Not well characterized

β_2_	Smooth muscle of afferent/efferent arterioles	Vasodilation and increases renal blood flow
	Apical and subapical compartment of proximal and distal tubular epithelia, podocytes	Currently being studied

β_3_	Arginine vasopressin sensitive nephron segments	Modulates sodium and water reabsorption contributing to volume and osmotic regulation

**Table 2:** In vitro studies showing the mechanisms by which β_2_-AR agonists produce beneficial effects.

S. No	Authors and year of publication	Beneficial mechanism postulated
1	Wills et al, 2012 [48]	Selective stimulation of β_2_-ARs by formoterol resulted in MB induction in renal proximal tubule cells.
2.	Arif et al., 2019 [49]	Formoterol increased the oxygen consumption of podocytes and thereby increased the recovery rate in injured podocytes. Fragmented mitochondria were restored with formoterol.
3	Cleveland et al., 2020 [45]	In diabetic kidney disease, there is mitochondrial dysfunction in renal proximal tubules due to increased mitochondrial fission and reduced fusion mediated by dynamin-related protein 1 (Drp 1) and mitofusin, respectively. Formoterol prevents the increase in Drp1 and restores the function of mitofusin 1.

**Table 3:** Animal studies showing the mechanisms by which β_2_-AR agonists produce beneficial effects.

S No	Authors and year of publication	Mechanism beneficial in diabetic kidney disease
1.	Wills et al., 2012 [48]	Formoterol exposure for 3 days increased mRNA levels of multiple genes involved in mitochondrial regulation and function in kidneys of male mice.
2.	Jesinkey et al., 2014 [50]	Administration of formoterol in mice with reperfusion injury has shown that formoterol induced MB, restored mitochondrial protein levels, promoted recovery following I/R induced injury, and decreased renal cell necrosis in mice.
3	Noh et al., 2017 [34]	Long term treatment with β_2_-AR agonist salbutamol normalized the renal changes such as the increased mesangial matrix area, higher expression of type I/IV collagen, and fibronectin exhibited by the Zucker diabetic fatty rat. Inflammatory mediators were reduced, and expression of beta arrestins was enhanced
4	Arif et al., 2019 [49]	In acute nephrotoxic serum nephritis and adriamycin-induced glomerular injury animal models, formoterol reduced albuminuria, focal atrophy and tubular dilatation, damage of podocyte foot processes, glomerular fibrosis, and sclerosis.
5.	Cleveland et al., 2020 [45]	In mouse models, formoterol prevented the increase in Drp1 and restored the function of mitofusin 1, which is increased due to high glucose in the early stage DKD. Similar changes were observed in in vitro settings.
6.	Arif et al., 2024 [51]	In high fat diet type 2DM and streptozotocin-induced T1 DM mice, formoterol facilitated recovery of diabetic kidney injury and reduced albuminuria and serum creatinine, and there was an increase in PGC-1 alpha in podocytes

## Data Availability

Data sharing is not applicable to this article as no datasets were generated or analyzed during the current study.
